# Effects of Antipsychotic Administration on Brain Glutamate in Schizophrenia: A Systematic Review of Longitudinal ^1^H-MRS Studies

**DOI:** 10.3389/fpsyt.2017.00066

**Published:** 2017-04-28

**Authors:** Alice Egerton, Akarmi Bhachu, Kate Merritt, Grant McQueen, Agata Szulc, Philip McGuire

**Affiliations:** ^1^Department of Psychosis Studies, King’s College London, Institute of Psychiatry, Psychology and Neuroscience, London, UK; ^2^Department of Psychiatry, Medical University of Warsaw, Warsaw, Poland

**Keywords:** schizophrenia, magnetic resonance spectroscopy, glutamates, antipsychotics, treatment response

## Abstract

Schizophrenia is associated with brain glutamate dysfunction, but it is currently unclear whether antipsychotic administration can reduce the extent of glutamatergic abnormality. We conducted a systematic review of proton magnetic resonance spectroscopy (^1^H-MRS) studies examining the effects of antipsychotic treatment on brain glutamate levels in schizophrenia. The Medline database was searched to identify relevant articles published until December 2016. Inclusion required that studies examined longitudinal changes in brain glutamate metabolites in patients with schizophrenia before and after initiation of first antipsychotic treatment or a switch in antipsychotic treatment. The searches identified eight eligible articles, with baseline and follow-up measures in a total of 168 patients. The majority of articles reported a numerical reduction in brain glutamate metabolites with antipsychotic treatment, and the estimated overall mean reduction of 6.5% in Glx (the combined signal from glutamate and glutamine) across brain regions. Significant reductions in glutamate metabolites in at least one brain region were reported in four of the eight studies, and none of the studies reported a significant glutamatergic increase after antipsychotic administration. Relationships between the degree of change in glutamate and the degree of improvement in symptoms have been inconsistent but may provide limited evidence that antipsychotic response may be associated with lower glutamate levels before treatment and a greater extent of glutamatergic reduction during treatment. Further longitudinal, prospective studies of glutamate and antipsychotic response are required to confirm these findings.

## Introduction

Animal, postmortem, and genetic studies indicate that schizophrenia is associated with abnormalities in glutamatergic neurotransmission (1), but it is unclear whether antipsychotic treatment may impact on glutamate dysfunction in patients. Our recent meta-analysis suggests that schizophrenia is associated with a general elevation in glutamate metabolites, with some variation observed across brain regions and patient subgroups (2). Elevations in frontal cortical or hippocampal glutamate may lead to secondary elevations in striatal dopamine release, characteristic of schizophrenia (3–6). Observations of glutamate dysfunction prior to illness onset (7–9), and in patients who are antipsychotic naïve or who have received minimal antipsychotic treatment (10, 11), suggest that glutamatergic dysregulation is a pathological feature of schizophrenia, rather than an effect of antipsychotic exposure. However, it is unknown whether antipsychotic treatment can reduce, or indeed worsen, glutamatergic abnormalities. Potentially, modulation of glutamatergic transmission with antipsychotic treatment could occur *via* downstream effects of D2 antagonism and/or *via* interactions at other receptor subtypes. Alternatively, if current antipsychotics do not adequately address glutamatergic dysfunction, this would support the suggestion that adjunctive treatment with glutamatergic agents may have additional therapeutic benefit.

The idea that antipsychotics may modulate glutamatergic neurotransmission is supported by experimental animal studies showing a reduction in frontal cortical glutamate following administration of some, but not all antipsychotics (12–19). In rodents, decreases in frontal glutamate have been observed using *ex vivo* proton nuclear magnetic resonance (^1^H-NMR) following administration of clozapine and olanzapine, but not haloperidol, risperidone, or aripiprazole (12). *In vivo* microdialysis studies in rodents have demonstrated reductions in pharmacologically induced elevations in frontal glutamate by risperidone, paliperidone, clozapine, aripipirazole, olanzapine, and haloperidol (13–19). However, a lack of significant effects of haloperidol on resting or stimulated glutamate metabolites in the rat brain have also been reported (19–22), and when antipsychotics are administered in the absence of pharmacologically stimulated glutamate release, antipsychotic-induced glutamate elevations may also be observed (23, 24). Together, these studies may suggest that antipsychotic glutamate-modulatory effects may be dependent on the animal model or glutamatergic assay, as well as the level of basal glutamatergic tone. Differential effects of antipsychotics may also be mediated by differing receptor binding profiles, and it has been suggested that downregulation of 5HT_2A_ receptors by antipsychotics with high 5HT_2A_ affinity may be important in reducing glutamatergic signaling (15, 16).

In man, brain glutamate levels can be measured *in vivo* using proton magnetic resonance spectroscopy (^1^H-MRS), usually within a specific *a priori* brain region of interest. Depending on the achieved resolution, ^1^H-MRS can provide concentration estimates for glutamate and glutamine or, at lower field strengths, the combined glutamate plus glutamine signal, which is termed Glx. The idea that antipsychotics may reduce glutamate elevation in schizophrenia is supported by a cross-sectional study that detected an elevation in Glx in non-medicated, but not in medicated schizophrenia (25). In our recent meta-analysis, meta-regression showed no significant relationship between regional glutamate, glutamine, or Glx and mean chlorpromazine equivalent antipsychotic dose (2). Nonetheless, longitudinal studies examining glutamate metabolites before and after antipsychotic treatment are required to address this question directly. Several such studies have now been published and the purpose of this article is to provide systematic review of their findings.

As a second objective, we also review the relationships between glutamate and symptomatic response in longitudinal studies of antipsychotic treatment. Cross-sectional ^1^H-MRS studies show that glutamate metabolite levels differ between patients who have or have not responded well to antipsychotic treatment (26–29). This may suggest that glutamate level may predict the degree to which symptoms are likely to respond to antipsychotic administration, or that symptom reduction occurs in parallel with antipsychotic glutamatergic modulation. We, therefore, appraised the evidence from longitudinal studies for the value of glutamate levels in predicting or monitoring antipsychotic response.

## Methods

### Study Selection

The review was conducted in accordance with PRISMA guidelines (30). The Medline electronic database was searched to identify journal articles published until 19 December 2016, using the following freeform and MeSH search terms: (“GLUTAMATE” OR “GLX”) AND (“SPECTROSCOPY” OR “MRS”) AND (“ANTIPSYCHOTIC”) AND (“SCHIZOPHRENIA” OR “PSYCHOSIS”). Reference lists of the returned articles were hand searched for further relevant publications.

Inclusion required that articles were published in peer-reviewed journals in English or English translation. Inclusion also required that studies reported ^1^H-MRS glutamate, glutamine, or Glx before and after either first initiation of antipsychotic medication or initiation of a change in antipsychotic administration. Inclusion was limited to investigations performed in patients with first episode psychosis, schizophrenia, or schizoaffective disorder. Where separate articles reported overlapping datasets, the article reporting the largest dataset was included. Where articles reported glutamate measures over multiple time-points, the glutamate measure at longest time-point was included to provide maximal time for any antipsychotic effects to emerge.

Returned articles were initially screened for inclusion through reading of article titles and abstracts. Full text was then screened for articles potentially meeting the inclusion criteria. Two authors independently performed the searches and identified articles for inclusion (Akarmi Bhachu and Alice Egerton).

### Data Extraction

For qualitative comparison of antipsychotic-induced change in glutamate metabolites across studies, the percentage difference in glutamatergic metabolite level over the antipsychotic treatment period was extracted from each article. This was calculated as the percentage mean difference (PMD), where PMD = [(mean glutamate metabolite level after treatment − mean glutamate metabolite level before treatment)/mean glutamate metabolite level before treatment] × 100. Where these values were not reported (31, 32), they were extracted from figures using WebPlotDigitizer (http://arohatgi.info/WebPlotDigitizer). Extracted data also included the reported statistical significance of the change in glutamatergic metabolite over the antipsychotic treatment period, the demographic and clinical characteristics of the sample, the antipsychotic/s prescribed and the duration of treatment, and the brain region investigated.

## Results

The initial search identified 51 articles, of which 40 were excluded at the title and abstract screening stage. At the full-text screening, one study was excluded as it was not available in English (33). Analysis of the sample reported in Ref. (34) was subsequently extended (35). One article (36) was excluded as it reported a subsample of participants included in a larger cohort and longer term follow-up (31). This resulted in final inclusion of eight original articles (10, 31, 32, 34, 37–40).

### Methodological Characteristics

The methodological characteristics of the included articles are provided in Table 1. Eight articles provided glutamate measures at baseline and after antipsychotic administration in a total of 168 patients. The sample sizes completing to ^1^H-MRS follow-up ranged from 7 to 42 patients. Three studies recruited patients with first episode psychosis who were antipsychotic naïve or had received minimal antipsychotic exposure (10, 31, 37). A further study in first episode psychosis did not describe antipsychotic exposure prior to baseline glutamatergic measurement (39). Two studies in chronic schizophrenia included washout periods of at least 7 days prior to baseline glutamate measurement and antipsychotic re-initiation (34, 40). One study in chronic schizophrenia investigated the change in glutamate measures following a switch from conventional antipsychotic treatment to olanzapine and did not include a washout period (38). The final study did not specify stage of illness, but included only patients who were antipsychotic naïve or antipsychotic-free for a minimum of 6 months (32).

**Table 1:** **Methodological characteristics of studies investigating the effects of antipsychotics on brain glutamate measures**.

Reference	*N*	Age	Illness stage	DOI	AP regime	AP	Pre-baseline AP	Months
(32)	34	NR	NR	NR	NR	Hal; Tfpz; Pzd; Clz	Naïve or >6 months w/o	1–6
(38)	14	NR	SZ	NR	Fixed	Ol	Conventional AP, no w/o	2
(40)	14	32 ± 7	SZ	9 ± 6	Fixed	Ri	>7 days	2
(10)	7	27 ± 9	FEP	0.6 ± 0.8	Flexible	Qu, Ri, Ar, Hal	Lifetime exposure <3 weeks	12
(31)	17	25 ± 7	FEP	1.8 ± 2	Flexible	Hal, Zpx, Ri, Ol, Qu, Zip; Clz	Naïve	80
(34)	42	32 ± 6	SZ	0.2 ± 0.5	Flexible	Ri, Ol, Clz, Pz, Cpz, Ppz	w/o >7 days	1.4–2.1
(39)	16	31 ± 12	FEP	NR	Flexible	Ri, Olz, Ar, Qu	NR	6
(37)	24	27 ± 8	FEP	0.4 ± 0.5	Fixed	Ri	Naïve	1

*Age, mean ± SD years; AP, antipsychotic; DOI, mean ± SD duration of illness in years; FEP, first episode psychosis; N, number of patients completing the follow-up assessment; NR, not reported; SZ, schizophrenia; w/o, washout period; Ar, aripiprazole; Clz, clozapine; Hal, haloperidol; Ol, olanzapine; Ppz, perperazine; Pz, perazinze; Pzd, pimozide; Qu, quetiapine; Ri, risperidone; Tfpz, trifluoperazine; Zip, ziprasidone; Zpx, zuclopenthixol*.

The majority of studies examined glutamatergic change in samples including patients on a variety of typical and atypical antipsychotic drugs, administered as standard clinical care (10, 31, 32, 34, 39) (Table 1). The remaining three studies investigated specific antipsychotic compounds, with one study investigating olanzapine (38) and two studies investigating risperidone (37, 40). The antipsychotic treatment period ranged from 1 to 80 months.

Four studies acquired glutamate measures at a field strength of 1.5-T and, therefore, reported glutamate primarily as the combined Glx signal (32, 34, 38, 40). Two studies were performed at 3-T, one of which reported Glx (39) and one of which reported both glutamate and Glx (37). The remaining two studies, performed at 4-T, reported glutamate, glutamine (10, 31), and Glx (31). Glutamatergic measures were reported as water-scaled values in ratio to voxel creatine (Cr) (32, 34, 38–40) or corrected for voxel cerebrospinal fluid (CSF) content (10, 31, 37). Brain regions investigated included the frontal cortex (seven studies), thalamus (four studies), temporal cortex (two studies), basal ganglia or striatum (two studies), parieto–occipital cortex (one study), and cerebellum (one study) (Table 2).

**Table 2:** **Results of studies investigating the effects of antipsychotics on brain glutamate measures and relationships with symptoms**.

Reference	Voxel location	Field strength (T)	Glutamate measure	PMD	Relationships with symptom change
(32)	FC	1.5	Glx/Cr	−21^a^^,^^b^	Positive correlation between ΔGlx and ΔBPRS score
(38)	R. ACC	1.5	Glx/Cr	+12	NS correlation between ΔGlx and ΔSANS total score. Sig. increase in Glx in responders (+46%) compared to non-responders (−21%)
	L. ACC				
(40)	L. FC	1.5	Glx/Cr	−7	NS correlation between ΔGlx and ΔPANSS
	L. TC			−16	
	L. Thal			0.5	
(10)	ACC	4	Glu; Gln; /CSF	NA	
	L. FWM				
	L. Thal				
(31)	L. ACC	4	Glx; Glu; Gln; /CSF	5, 5, 4	Positive correlation between ΔGlx in L. Thal and Life Skills Profile Rating Scale score at follow-up
	L. Thal		Glx; Glu; Gln; /CSF	−9^a^,−1, −16	
(34)	L. FC	1.5	Glx/Cr	−6	NS correlation between ΔGlx in temporal lobe and ΔPANSS
	L. TC			−15^a^	
	L. Thal			2	
(39)	FC	3	Glx/Cr	−27^a^	NS correlations between ΔGlx and ΔPANSS
	L. BG			−14	
	POC			−5	
(37)	R. Striatum Cblm	3	Glx; Glu; /CSF	−5; −7^a^	Negative correlation between both ΔGlx and ΔGlu in R. striatum and ΔPANSS general score
			Glx; Glu; /CSF	+9; +3	

*ACC, anterior cingulate cortex; BG, basal ganglia; BRPS, Brief Psychiatric Rating Scale; Cblm, cerebellum; Cr, scaled to voxel creatine; CSF, corrected for voxel cerebrospinal fluid content; Δ, change; L, left; FC, frontal cortex; FWM, frontal white matter; Gln, glutamine: Glu, glutamate; Glx, glutamate plus glutamine; NA, not available; NS, non-significant; PANSS, positive and negative syndrome scale for schizophrenia; PMD, percentage mean difference; POC, parieto–occipital cortex; R, right; SANS, scale for assessment of negative symptoms; TC, temporal cortex; Thal, thalamus*.

*^a^Reported as significant finding*.

*^b^Significance calculated from figure in article*.

### Effect of Antipsychotic Treatment on Brain Glutamate Levels

**Figure 1:**
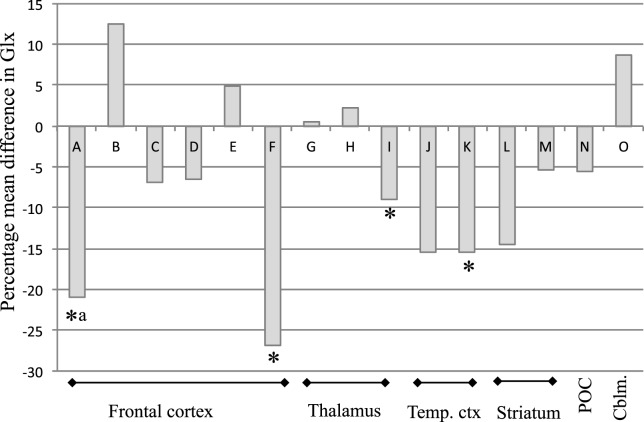
**Percentage mean difference (PMD) in Glx in individual studies of Glx at baseline and after antipsychotic administration in schizophrenia**. PMD was calculated as [(mean Glx level at after treatment−mean Glx level before treatment)/mean Glx level before treatment] × 100. Abbreviations: Glx, combined signal from glutamate and glutamine; Temp. ctx, temporal cortex; POC, parieto–occipital cortex; Cblm, cerebellum. Letters relate to articles as follows: A (32) B (38) C (40) D (34) E (31) F (39) G (40) H (34) I (31) J (40) K (34) L (39) M (37) N (39) O (37). *Reported as significant finding; ^a^Significance calculated from figure in article.

Across brain regions, the PMD in Glx after antipsychotic treatment ranged from a 12.5% increase (38) to a 27% decrease (39). In the majority of observations (10 out of 15), there was a numerical reduction in Glx, with an overall mean decrease of 6.5% (Figure 1). The PMD in Glx could not be calculated from the information available in one article (10). A decrease in Glx of 6.5% (±11%) can be estimated to be associated with an effect size of approximately *d*_z_ = 0.6. Accordingly, this translates to a within-subjects’ sample size of 24 patients to observe a significant change in Glx over antipsychotic treatment, at 80% power and α = 0.05, two-tailed.

Four of the eight included studies reported statistically significant glutamatergic reductions over antipsychotic treatment in at least one brain region investigated (31, 34, 37, 39). Specifically, significant reductions in Glx occurred in the thalamus over 80 months of mixed antipsychotic administration (31), in the left temporal lobe over 2 months of mixed antipsychotic administration (34), and in the frontal lobe over 6 months of mixed antipsychotic administration (39), and in glutamate in right striatum over 1 month of risperidone administration (37). Additionally, Choe et al. (32) also reported a reduction in prefrontal cortical Glx over 1–6 months antipsychotic treatment in 29/34 patients but did not report the statistical outcome associated with this finding. Extraction and analysis of data from the figure provided in the article found a significant reduction in frontal cortical Glx [mean ± SD Glx at baseline: 0.95 ± 0.30; follow-up: 0.75 ± 0.31; paired samples *t*-test: *t*(29) = 3.06; *P* = 0.005]. There were no reports of significant increases in glutamate metabolites with antipsychotic treatment. Three of the eight studies did not report significant effects of antipsychotic treatment on glutamate measures in any brain area across the patient sample (10, 38, 40).

### Relationships between Change in Glutamate and Symptom Reduction on Antipsychotic Administration

The included studies reported mixed findings regarding associations between the degree of change in glutamate and the degree of symptomatic reduction with antipsychotic treatment (Table 2). Significant positive associations were reported for change in frontal Glx and change in the total score on the Brief Psychiatric Rating Scale (BPRS) (32), and for change in striatal glutamate and Glx and change in Positive and Negative Syndrome Scale (PANSS) general symptom score (37). One study reported a positive correlation between the change in thalamic Glx over 80 months antipsychotic treatment and Life Skills Profile Rating Scale Score at 80 months (31). These observations suggest that a greater degree of glutamate reduction is associated with a greater degree of symptomatic improvement during antipsychotic treatment. However, four of the eight included studies reported no significant correlations between changes in glutamate metabolite levels and changes in symptom severity (34, 38–40). While one of these studies found no significant correlation between change in glutamate and change in the Scale for Assessment of Negative Symptoms (SANS) total score on olanzapine administration (38), a secondary analysis dividing the group into responders and non-responders based on the change in SANS total score found a 46% Glx increase in responders, compared to a 21% Glx decrease in non-responders, and a significant difference between these groups (38). This is broadly inconsistent with the reports of positive associations between glutamate reduction and symptom reduction (32, 37).

None of the studies included in the review examined whether glutamate measures before antipsychotic treatment predicted the degree of subsequent symptomatic response to antipsychotic administration. However, a separate article (35) presenting additional analysis of data presented in a previous article (34) found that frontal Glx at baseline was significantly lower in subsequent responders than non-responders.

## Discussion

The main finding of this review is that the majority of studies reported a numerical reduction in glutamate metabolites following antipsychotic treatment in schizophrenia, with half of the reviewed studies finding significant reductions in at least one brain region. In contrast, no significant increases in glutamate metabolites were reported. Schizophrenia is associated with a general increase in glutamate metabolites, which varies with region and with illness stage (2). This review provides some suggestion that antipsychotics may reduce glutamatergic elevations in schizophrenia but indicates that this effect may be relatively small or limited to subgroups of patients.

The mean change in Glx over antipsychotic treatment was estimated from the available data as a decrease in the range of 6.5% across regions, which would require a within-subjects’ sample size of 24 patients to achieve 80% power. Only three of the eight available studies had sample sizes of 24 or more patients (32, 34, 37) and the three studies with the smallest sample sizes (10, 38, 40) were the studies that did not find significant effects of antipsychotics on glutamatergic measures, suggesting that they may have been underpowered. Two of these smaller studies (38, 40) also differed in that they recruited patients with long antipsychotic medication histories, which may have also limited the ability to observe further glutamatergic reduction.

Our estimates of percentage reduction in glutamatergic metabolites following antipsychotic treatment in schizophrenia and the associated sample size calculations are limited by the availability of relatively few published studies. These studies have also investigated different brain regions, and glutamate dysfunction in schizophrenia may vary by region as well as by illness stage (2). In some cases, it was also necessary to extract values from published figures, which is less accurate than using reported values, and measures included both creatine-scaled or voxel CSF corrected data. As further studies become available, estimates of the percentage reduction can be updated and formal meta-analyses of effect sizes can be performed including inspection of potential influences such as regional specificity, patient subgroups, and duration of antipsychotic treatment. General limitations of ^1^H-MRS include the estimation of the total concentrations of glutamate metabolites in the voxel, rather than imaging of glutamate involved in neurotransmission specifically, and inability to resolve glutamate from glutamine at lower field strengths and thereby interpretation of the combined Glx signal. Advanced methodological approaches, such as ^1^H-MRS at high field strengths or ^13^C-MRS to inspect glutamate cycling may address some of these issues in future studies.

Reductions in glutamate metabolites following antipsychotic treatment would be consistent with the cross-sectional report of elevated prefrontal cortical Glx in non-antipsychotic medicated but not antipsychotic-medicated schizophrenia (25). While the mechanism by which antipsychotics might reduce glutamate is not yet clear, rodent studies also find reductions in basal or stimulated glutamate following administration of some antipsychotics (12–19), which may relate to the 5HT_2A_ antagonist activity of atypical agents (16, 41, 42). Atypical antipsychotics may also indirectly modulate glutamate release by modifying glutamatergic receptor activity or density (43–45). The majority of patients included in the reviewed studies were prescribed atypical antipsychotics, which may, therefore, have increased the potential to observe glutamatergic reductions. Future studies could specifically compare the ability of D2-selective antipsychotics to antipsychotics with marked 5HT_2A_ affinity to modulate glutamate in schizophrenia.

The timescales over which antipsychotic effects on glutamatergic measures have been evaluated range from 4 weeks (37) to 7 years (31). It might be predicted that longer durations of antipsychotic treatment may be associated with progressive glutamate reductions. Across the included studies, there was not suggestion of this, for example significant decreases in frontal Glx were observed after administration of antipsychotics for 1–6 months (32, 39), but not 12 or 80 months (10, 31). Individual studies that included repeated glutamatergic measurement also do not evidence progressive glutamatergic reduction (10, 31). This may suggest that antipsychotic effects on glutamatergic systems are most apparent within the initial stages of treatment, which could be explored in future studies. A further consideration for longitudinal assessment of glutamatergic measures in schizophrenia is the potential interacting effects of other confounds, such as disease progression, aging effects, or volumetric changes. The study of longest duration detected correlations between the reductions in thalamic glutamine and gray matter loss in the superior temporal gyrus over 30 months (36), and gray matter loss in frontal, parietal, temporal, and limbic regions over 7 years (31), possibly reflecting excitotoxic processes.

The second aim of this article was to review the potential relationships between glutamate and the degree of antipsychotic response in longitudinal studies. Our review only identified one study prospectively examining the relationship between baseline glutamate and subsequent response, which reported higher baseline Glx/Cr across voxels in the frontal and temporal lobes and thalamus in subsequent antipsychotic non-responders than responders (35). Two studies also reported correlations between the extent of glutamatergic reduction and the extent of symptomatic improvement over the antipsychotic treatment period (32, 37). These findings are consistent with observations of higher frontal glutamate levels in some (26, 27, 46), but not all (29), cross-sectional studies comparing treatment-responsive and non-responsive schizophrenia, and of higher striatal Glx/Cr in treatment resistant compared to treatment-responsive patients (29). The observation that glutamate levels may predict antipsychotic response (35) is also supported by recent pharmacogenomic findings that single nucleotide polymorphisms in genes encoding glutamatergic proteins associate with response to risperidone in first episode psychosis (47). In contrast, the conflicting result of an increase in frontal Glx/Cr in olanzapine responders compared to non-responders was reported in one study (38), and several studies reported no correlations between glutamatergic change and symptomatic improvement (34, 38–40). The idea that antipsychotic administration is less effective in reducing glutamate and improving symptoms in patients with the highest levels of glutamate before treatment could be further explored through re-analysis of existing longitudinal ^1^H-MRS studies, and investigated in future longitudinal studies potentially combining glutamate ^1^H-MRS with glutamate genetic approaches.

## Author Contributions

AE and AB performed the literature searches and data extraction. KM and GM checked data for accuracy. All authors contributed to interpretation of the data and the final draft of the manuscript.

## Disclaimer

The views expressed are those of the authors and do not necessarily represent those of the NHS, NIHR, or Department of Health.

## Conflict of Interest Statement

The authors declare that the research was conducted in the absence of any commercial or financial relationships that could be construed as a potential conflict of interest.
